# Predictors of care home resident conveyance to hospital or referral to community pathways by a regional ambulance service attending medical emergencies: a retrospective cross sectional study

**DOI:** 10.1186/s13049-024-01294-y

**Published:** 2024-11-27

**Authors:** Aloysius Niroshan Siriwardena, Vanessa Botan, Graham Law, Despina Laparidou, Viet-Hai Phung, Ffion Curtis, Gregory Adam Whitley, Joseph Akanuwe, Elise Rowan, Rachael Fothergill, Susan Bowler, Maria Kordowicz, Nicoya Palastanga, Lissie Wilkins, Robert Spaight, Elizabeth Miller, Adam Gordon

**Affiliations:** 1 Community and Health Research Unit, School of Health and Social Care, Lincoln Medical School, University of Lincoln, Lincoln, LN6 7TS UK; 2 School of Social Sciences, Nottingham Trent University, Nottingham, NG1 4FQ UK; 3 Liverpool Reviews & Implementation Group (LRiG), University of Liverpool, Liverpool, L69 3GB UK; 4 East Midlands Ambulance Service NHS Trust, Cross O‘Cliff Court, Bracebridge Heath, Lincoln, LN4 2HL UK; 5 Lincolnshire Partnership NHS Foundation Trust, Lincoln, LN1 1FS UK; 6 Healthier Aging Patient and Public Involvement Group, University of Lincoln, Lincoln, LN6 7TS UK; 7 University of Nottingham, Nottingham, NG7 2RD UK

## Abstract

**Background:**

Care home residents are at higher risk compared with community dwelling elders for medical emergencies, often resulting in ambulance attendance and conveyance to hospital. We aimed to determine the factors predicting care home resident conveyance to hospital or referral to community pathways by an ambulance service.

**Methods:**

We used a retrospective cross-sectional study design analysing routine data from electronic clinical records from East Midlands Ambulance Service NHS Trust (EMAS). Data comprised all patients including care home residents attended by ambulance from 2018 to 2021. A multivariable logistic regression model was used to identify the main predictors of conveyance to hospital or referral to community services.

**Results:**

Data included 170,612 attendances to care homes representing 7.5% of the total number of EMAS attendances between 2018 and 2021. The main predictors of conveyance to hospital were being male (Relative Risk Ratio [RRR] 1.07, 95% Confidence Interval [CI] 1.03–1.10, *p* < 0.001), aged 70–79 years (RRR 1.09, 95%CI 1.03–1.17, *p* < 0.001) or 80–89 years (RRR 1.10, 95%CI 1.03–1.17, *p* < 0.001), situated in an area of higher deprivation (RRR 1.06, 95%CI 1.03–1.09, *p* < 0.001), or having dispatch categories which included cardiovascular (RRR 11.29, 95%CI 10.43–12.22, *p* < 0,001), trauma such as falls (RRR 9.50, 95%CI 8.97–10.05, *p* < 0,001) or neurological conditions (RRR 9.06, 95%CI 8.42–9.75, *p* < 0,001). Calls made by health care professionals (HCPs) (RRR 15.37, 95%CI 13.41–17.62, *p* < 0,001) or where patients had a higher National Early Warning Score (NEWS2) (RRR 1.23, 95%CI 1.22–1.24, *p* < 0,001) resulted in significantly increased conveyance.

**Conclusions:**

Various factors significantly predicted conveyance of care home residents to hospital by ambulance. These included HCP referral and a higher NEWS2 score confirming that severity of clinical condition of the patient significantly increased conveyance. Future interventions to prevent or address certain conditions such as falls or provide enhanced care in care homes may prevent some emergencies or reduce the likelihood of conveyance to hospital.

**Supplementary Information:**

The online version contains supplementary material available at 10.1186/s13049-024-01294-y.

## Background

Medical emergencies in residential or nursing care homes commonly occur and often result in calls to Emergency Medical Services (EMS) or general practitioners (GPs) [1] An ambulance EMS response usually results in conveyance to the Emergency Department (ED) and admission, leading to high costs and risks of hospitalisation [1]. The incidence of hospital transfer from care homes varies between countries, with one systematic review (13 studies from 7 countries) reporting at least 30 transfers for every 100 residents annually from residential care to ED [2]. Care home residents account for just 2.8% of the total elderly population (> 65 years old) in England but 6.5% of ED attendances [1].

Many care home residents transferred by ambulance to the ED are admitted, with almost half of nursing home residents hospitalised in one study, which was almost twice that of community dwellers after adjusting for age, sex, and ethnicity [3]. Those who were hospitalised had higher mortality compared with community dwelling elders [3]. Hospital transfer may be preventable in some cases, where “an existing condition would have been managed optimally in the NH [nursing home] at an earlier stage or when adequate prevention would have avoided its initial presentation” [4]. Although definitions of appropriateness and potential preventability of transfers are contested [4], over a half of emergency transfers to hospital are deemed potentially preventable using international criteria [5]. 

The decision to transfer to ED is important because of the risks and costs of preventable admission, with transfers sometimes occurring because of factors linked to patients (or their relatives) and providers [2, 6, 7]. EMS provider factors include unclear expectations of ambulance staff, lack of staff capacity or capability, and limited access to multidisciplinary support or problems communicating with primary care decision-makers [8]. Frontline ambulance staff faced with a resident’s worsening condition, insistence by family members or recommendation by a physician may consider a transfer to be unavoidable [9]. Nursing home characteristics, such as staff-resident ratio and skills [10] or advanced care planning and support from local health services, may also affect emergency care and reduce hospital transfers [11]. Previous studies have found that relatives’ concerns about nursing home care, lack of advance care planning or preparation for end of life, and goals of care may be factors in transfer decisions [12]. Contextual factors might include time of day or day of week, with most transfers from nursing homes to EDs taking place during weekday working hours [13]. 

Less is known about the detailed demographic factors related to care homes and residents attended, their clinical condition and physiological measurements which are associated with conveyance to hospital. In one study from Australia the odds of conveyance were higher for rural care homes, and residents with conditions such as depression, cardiovascular disease or osteoporosis, or prescribed antipsychotic or antidepressant medication [14]. In another study from Germany male care home residents with a higher care dependency and on multiple drugs were more likely to need acute care [15]. 

We aimed to investigate demographic and clinical predictors of ambulance conveyance to hospital or referral to community pathways (i.e. primary care, community nursing, rehabilitation or related services) following an emergency call from a care home. Demographic factors of interest include age, sex, socioeconomic deprivation and rurality while clinical factors included clinical condition (at dispatch and on scene), call urgency and physiological observations. We hypothesised that older age, male sex, higher socioeconomic deprivation and rurality, more urgent clinical conditions, greater call urgency and more abnormal physiological observations would be associated with higher rates of conveyance or referral.

## Methods

### Study design and setting

We used a retrospective cross-sectional study design to explore associations between demographic and clinical factors and ambulance conveyance to hospital or referral to community pathways. Routine data were collected for analysis from the electronic clinical records accessed from the East Midlands Ambulance Service (EMAS) between January 1, 2018, and December 31, 2021.

EMAS is one of 10 ambulance services in England UK, covering 6500 square miles of rural, urban and coastal areas, serving a population of 4.8 million residents, responding to around 3500 calls a day. During the study period, the attending ambulance clinicians’ scope of practice varied depending on their clinical rank, with paramedics having a greater scope of practice with more autonomy, and Emergency Medical Technicians having a more restricted scope of practice that sometimes required discussion with a senior clinician to discharge a patient on scene. Both ranks utilised nationally agreed [16] and local guidelines to inform their conveyance decision making. An example includes the use of the National Early Warning Score 2 (NEWS2), a simple aggregate scoring system in which a score is allocated to physiological measurements [17], ranging from 0 to 20. Ambulance clinicians utilised the Paramedic Pathfinder tools [18] which advised that for patients aged 16 years and above with a medical illness and a NEWS2 score of 5 or more, or 3 in a single parameter, they should consider either transport to hospital, a specialist pathway, or discussion involving senior clinical advice.

#### Ethical approval

was obtained from the UK Health Research Authority (Reference 21/WM/0229).

### Data collection and processing

Data were obtained from EMAS and included information about unique, anonymised patient number, call number, call (urgency) category, clinical condition (‘chief complaint’ recorded through the Computer-Aided-Dispatch [CAD] system and clinical ‘impression’ recorded on scene by ambulance clinicians), date and time of call, geographical location (incident postcode), demographic information (age, sex, and ethnicity), times (time to arrive, time spent on scene), conveyance, first vital signs (heart rate, respiratory rate, blood pressure, oxygen saturation, and temperature), Glasgow Coma Scale (GCS), and the National Early Warning Score (NEWS2) [17]. 

Implausible vital sign measurements were censored as follows: respiratory rate higher than 120 breaths per minute and temperature below 12 and above 47 degrees [19]. NEWS2 scores were calculated from the dataset for all participants based on physiological parameters (vital signs) according to the NEWS2 scoring system [17]. NEWS2 scores were also calculated and used by paramedics at the time of patient contact but these were not used in the analysis. The impression on scene variable had been grouped in eight clinical categories by clinicians at EMAS: cardiovascular, respiratory, trauma, neurological, mental health, gynaecological, medical, and other (examples of conditions present in each category can be found in Supplementary Table 1).

Attendances to care homes were identified based on postcode and name of care home provided by EMAS after linking this data with the list of care homes in the East Midlands region. Ethnicity was recategorised into five groups as follows: White (e.g. “White British,” “White Irish,” or “any other White background”), Black (e.g. “Black”, “Ghanian”, “Somali”, “Any other black background”), South-East Asian (e.g. “Chinese”, “Filipino”, “Thai”), Asian ( e.g. “Indian” ,“Pakistani”, “Iraqi, ”Afghan”), and mixed (e.g. “White and Black African,” “White and Asian,” or “Any other mixed background”). Subject age was obtained after subtracting patients’ date of birth from date of call and divided into six categories: under 60, 60–69, 70–79, 80–89, 90–99, and over 100 years. Rurality (rural versus urban), and deprivation levels (Index of Multiple Deprivation (IMD) [20] ranging from 1 – most deprived to 10 – least deprived) were established based on the incident postcode after linking ambulance with the Office of National Statistics (ONS) data. Deprivation was further categorised into high (IMD 1–5) and low (IMD 6–10) deprivation. Conveyance was divided into three main categories: conveyed, not conveyed, and referred to community pathways.

### Statistical analysis

Descriptive statistical analyses including frequencies and proportion tests were used to compare care home ambulance attendances with non-care home ambulance attendances. Non-parametric Wilcoxon tests were used to compare ambulance arrival times and times spent on scene for care homes and the rest of the sample. A multinominal multivariable logistic regression analysis was conducted using conveyance as the main outcome and sex, age, deprivation, rurality, clinical condition (clinical ‘impression’ recorded by ambulance staff), and NEWS2 scale or its component physiological variables as predictors. We estimated a minimum sample (N) of 1000 cases for the multivariable regression model based on the number of independent variables (k up to 10) and smallest proportion (p) of conveyances or referrals (p at least 10%) according to the formula *N* = 10k/p [21]. The cases of cardiac arrest which had values of 0 for all vital signs were excluded from the analysis after checking that the restricted sample model which excluded them represented a better fit which explained 13% of the variation in the data (Χ^2^ = 21,501.25, *p* < 0.001, R^2^ = 0.13) compared to the full sample which explained 12% (Χ^2^ = 19,919.12, *p* < 0.001, R^2^ = 0.12). Multiple imputation for data missing at random (MAR) was used to impute missing values for condition categories [22]. A multivariable logit model using Stata *mi function* with five iterations was applied to impute missing data using age, gender, call category, and NEWS2 score as predictors [23]. The multinominal multivariable regression analysis run on the sample without imputed data confirmed the same significant predictors of conveyance as seen in Supplementary Table S2. All analyses were conducted using Stata 17.0.

## Results

### Descriptive statistics

Overall, there were 170,612 attendances to care homes representing 7.5% of a total of 2,288,304 attendances occurring over a period of four years.

When compared to the rest of the sample, ambulance attendances to care homes were predominantly for older patients (median age in years = 86, IQR: 78–91 versus median age = 65 years, IQR:39–81], females (60.0% versus 52.0%), and patients of White ethnicity (98.1% versus 93.0%). Detailed results of patients’ demographics can be seen in supplementary materials and results S3.

The most frequent conditions recorded by clinicians as impression on scene where ambulances attended care homes were head injury (9.3%) and chest infections (8.3%) (Table 1). The most frequent conditions recorded on the CAD system as chief complaints were falls, representing 11.3% of total attendances to care homes, and suggesting that head, limb, and other injuries were most likely caused by falls. Detailed results of conditions as recorded by chief complaints are in Supplementary Table S3.

**Table 1:** Numbers and percentages of clinical conditions in patients most frequently attended by ambulance services in care homes compared with non-care home attendances

Condition	Care home attendances	Non-care home attendances
Head injury **	15,483 (9.28%)	61,441 (3.01%)
Chest infection**	13,889 (8.32%)	105,281 (5.15%)
Limb injury	7,553 (4.52%)	66,688 (3.26%)
Collapse	7,098 (4.25%)	65,888 (3.22%)
Other infection	5,768 (3.46%)	48,632 (2.38%)
Other respiratory problem	4,806 (2.88%)	74,687 (3.65%)
Acute abdominal problem*	4,799 (2.87%)	120,208 (5.88%)
Sepsis	4,687 (2.81%)	19,295 (0.94%)
No apparent problem	4,667 (2.80%)	48,631 (2.38%)
Fall non-injury	4,511 (2.70%)	33,572 (1.64%)

*p < 0.05; ** *p* < 0.001

Ambulance attendance to care homes were less frequent to category 2 calls (more urgent) and more frequent to category 3 calls (less urgent) compared to non-care home attendances (Table 2).

**Table 2:** Numbers and percentages of call categories attended by ambulance services in care homes compared with non-care home attendances

Call Category	Care home attendances	Non-care home attendances
1 (most urgent)	16,200 (9.51%)	244,451 (11.56%)
2**	94,000 (55.20%)	1,359,106 (64.28%)
3**	43,478 (25.53%)	403,693 (19.09%)
4	8,420 (4.94%)	56,372 (2.67%)
5 (least urgent)	831 (0.49%)	13,476 (0.64%)
Healthcare professional call	7,367 (4.33%)	37,415 (1.77%)

*p < 0.05; ** *p* < 0.001

Ambulances took approximately one minute longer to arrive to care homes. Median arrival time to care homes was 10.23 minutes [5.6, 17.55] and in the rest of the sample was 9.17 minutes [5.33, 15.60] (W = 1.9136, *p* < 0.001). Ambulances spent approximately five minutes longer time on scene for care home attendances. Median time spent on scene in care homes was 40.68 minutes [IQR: 30.78, 53.73] and in the rest of the sample was 35.77 minutes [IQR: 25.80, 49.50] (W = 9.6695, *p* < 0.001).

### Predictors of conveyance to hospital from care homes

The main predictors of conveyance to hospital were being male (Relative Risk Ratio [RRR] 1.07, 95% Confidence Interval [CI] 1.03–1.10, *p* < 0.001), aged 70–79 years (RRR 1.09, 95%CI 1.03–1.17, *p* < 0.001) or 80–89 years (RRR 1.10, 95%CI 1.03–1.17, *p* < 0.001), situated in an area of higher deprivation (RRR 1.06, 95%CI 1.03–1.09, *p* < 0.001), or having clinical impression categories which included cardiovascular (RRR 11.29, 95%CI 10.43–12.22, *p* < 0,001), trauma such as falls (RRR 9.50, 95%CI 8.97–10.05, *p* < 0,001) or neurological conditions (RRR 9.06, 95%CI 8.42–9.75, *p* < 0,001) (Table 3). Calls made by health care professionals (HCPs) (RRR 15.37, 95%CI 13.41–17.62, *p* < 0,001) or where patients had a higher National Early Warning Score (NEWS2) (RRR 1.23, 95%CI 1.22–1.24, *p* < 0,001) resulted in significantly increased conveyance.

**Table 3:** Predictors of conveyance and referral in care homes. Baseline category for each variable is shown in brackets

Baseline (Not conveyed)	Conveyed to ED		Referred to a community pathway	
	**RRR**	**95% CI**	RRR	95% CI
**Sex (Female)**	1	-	1	-
Male	1.07**	1.03, 1.10	0.97	0.91, 1.03
Transgender	2.19	0.98, 4.87	0.58	0.07, 4.66
**Age (under 60)**	1	-	1	-
60–69	1.05	0.96, 1.14	1.19	1.00, 1.42
70–79	1.09**	1.03, 1.17	1.38	1.20, 1.59
80–89	1.10**	1.03, 1.17	1.59**	1.40, 1.80
90–99	0.98	0.92, 1.04	1.84**	1.62, 2.09
100 and over	0.61	0.54, 0.70	1.88**	1.51, 2.33
**Deprivation (Low)**	1	-	1	-
High	1.06**	1.03, 1.09	0.97	0.92, 1.03
**Rurality (Rural)**	1	-	1	-
Urban	1.01	0.98, 1.05	1.22**	1.13, 1.30
**Impression Group (Other)**	1	-	1	-
Medical	8.93**	8.46, 9.42	3.79**	3.45, 4.17
Gynaecological	23.84**	15.37, 36.99	4.36**	2.01, 9.49
Mental Health	3.25**	2.93, 3.60	3.08**	2.60, 3.66
Neurological	9.06**	8.42, 9.75	2.78**	2.43, 3.18
Trauma	9.50**	8.97, 10.05	1.54**	1.37, 1.73
Respiratory	6.81**	6.35, 7.30	3.67**	3.26, 4.13
Cardiovascular	11.29**	10.43, 12.22	1.93**	1.64, 2.27
**Call Category (1)**	1	**-**	1	**-**
2	1.48**	1.39, 1.57	1.18**	1.07, 1.31
3	1.22**	1.14, 1.30	0.86*	0.77, 0.96
4	13.28**	11.48, 15.35	1.06	0.76, 1.48
5	1.05**	0.79, 1.41	0.39*	0.17, 0.90
HCP	15.37**	13.41, 17.62	1.11	0.83, 1.50
**First NEWS2**	1.23**	1.22, 1.24	1.11**	1.10, 1.12

*p < 0.05; ** *p* < 0.001

The main predictors of referral were being in an urban location (RRR 1.22, 95%CI 1.13, 1.30, *p* < 0,001), gynaecological (RRR 4.36, 95%CI 2.01, 9.49, *p* < 0,001), medical (RRR 3.79, 95%CI 3.45, 4.17, *p* < 0,001), respiratory (RRR 3.67, 95%CI 3.26, 4.13, *p* < 0,001), and mental health clinical impression categories (RRR 3.08, 95%CI 2.60, 3.66, *p* < 0,001). Call category 2 (RRR 1.18, 95%CI 1.07, 1.31, *p* < 0,001) and NEWS2 (RRR 1.11, 95%CI 1.10, 1.12, *p* < 0,001) score also increased the likelihood of being referred. Each point increase in NEWS2 carried a 1.23 increased RRR of being conveyed or 1.11 for being referred.

The multivariable logistic regression analysis without imputed data confirmed the same significant predictors of conveyance (Supplementary Table 4). A multivariable logistic regression model was run using physiological parameters instead of NEWS scale (Supplementary Table 5).

## Discussion

### Main findings

Ambulance attendances to care homes represented 7.5% of all attendances occurring over the four-year period 2018–2021. Compared to the non-care attendances, ambulance attendances to care home residents were significantly greater for older (median age = 86 years vs. 65 years), female (60.0% versus 52.0%) patients of White ethnicity (98.1% versus 93.0%). The most frequent conditions identified on attendance by ambulance clinicians were falls representing 11.3% of total attendances to care homes with the more frequently occurring clinical categories of head, limb, and other injuries therefore most likely caused by falls.

The main predictors of conveyance to hospital were being male, aged 70–89 years, resident in a care home located in an area of higher deprivation, or having clinical impression categories which included cardiovascular, trauma such as falls, or neurological conditions. Calls made by health care professionals or where patients had a higher National Early Warning Score resulted in significantly higher rates of conveyance. The main predictors of referral to primary care or alternative pathways of care were care homes in an urban location, and calls for residents with gynaecological, medical, respiratory, and mental health conditions. Call category 2 and a higher NEWS2 score also increased the likelihood of being referred.

### Comparison with previous research

In an interrupted-time series analysis, using the same dataset, we compared total numbers of ambulance attendances to care homes per week before and during the Covid-19 pandemic considering the three UK national lockdown periods while including seasonality (i.e., month of the year), call category, deprivation, and rurality as covariates in the model. We found significant reductions in ambulance attendances to care homes in the first and third lockdowns but not the second. and rates remained lower during the pandemic compared to pre-pandemic levels [24]. 

Few other studies have explored predictors of emergency hospitalisation in care home residents. In one study from Victoria, Australia the odds of conveyance were higher for care home residents in rural areas, with a history of depression, cardiovascular disease or osteoporosis, and those prescribed antipsychotic or antidepressant medication whereas residents who had fallen were less likely to be transferred than those with a medical complaint [14]. In a study from Germany male care home residents with a higher care dependency and on multiple drugs were more likely to need acute care [15]. 

In relation to age and sex or gender, a previous systematic review found that male care home residents were more likely to be transferred to the ED, although the reasons for this were unclear and there was no clear relationship between age and conveyance [25]. In another cross-sectional study from Germany exploring age and sex of residents transferred to hospital, where 43.1% were hospitalised at least once during the preceding 12 months, the rate of transfer was greater in those institutionalised more recently, within 6 months compared to those with a longer length of stay (65.7% vs. 39.5%). Males were more likely to be hospitalised than females (52.4% vs. 40.3%) but this was not statistically significant. In females, the chance of being hospitalized decreased steadily with age whereas older males aged over 85 years compared with those aged 75–84 years were more likely to be transferred [26]. 

A systematic review of observational studies of prehospital early warning scores have shown that very low and high scores were able to discriminate between patients who are not or who are likely to deteriorate [27] and as these scores tend to be used by ambulance clinicians to determine the severity of a patient’s condition it is perhaps not surprising that we found higher rates of transfer for higher NEWS2 scores. NEWS scores have been used in care homes and by care home staff during the COVID-19 pandemic for surveillance [28] and have been found to be helpful for assessing deterioration in residents’ conditions prior to calling an ambulance [29]. The clinical decision making to convey a patient to hospital or not is a complex aggregate of multiple sources of information, clinical experience, and patient wishes, therefore it is unlikely that ambulance clinicians would have based their conveyance decision based on the NEWS2 score alone, as for example, patients receiving palliative care or at the end of life often have abnormal NEWS2 scores but this may not necessitate transport to hospital.

The link with socioeconomic deprivation is novel. It is less surprising that a healthcare professional calling an ambulance, was more likely to result in conveyance, where their clinical assessment informed the decision to call an ambulance and where a clinical recommendation, most likely to be from a physician, to convey would be unlikely to be overturned by ambulance staff.

A previous systematic review of interventions to reduce unplanned admissions from care homes, which included 124 publications or reports (of which 30 were from the UK), found low quality evidence from uncontrolled study designs with small sample sizes, but suggested that that quality improvement programmes integrating health and social care to provide additional support to care homes and advance care planning could reduce unplanned admissions, whereas staff training and more specific interventions, for example medication reviews, showed mixed results [30]. 

### Strengths and limitations

Despite the large dataset of routine administrative and clinical data with high rates of recording and low levels of missing data, limitations included that dispatch and clinical impression codes were selected from existing categories. We did not have access to hospital admission, inpatient or discharge data so clinical diagnoses may have changed subsequently. The retrospective observational design was a limitation in that unmeasured or unknown confounders could not be excluded and the associations found do not imply causation.

### Implications for policy, practice, and research

Our findings provide useful information for care home staff and ambulance crews on clinical conditions and clinical severity which increased the likelihood of conveyance. This has implications for prevention and assessment, the training of care home and ambulance staff, and the design of interventions to improve care of emergency conditions developing in care home residents. Interventions to prevent or address certain conditions such as falls or to provide enhanced care in care homes may prevent some emergencies or reduce the risk of conveyance to hospital.

Future research may include using a larger, more representative dataset involving a greater number of ambulance services to develop a valid model for transfer to hospital. Development of interventions involving care homes, ambulance staff, primary care and acute services to improve pathways of care and communication between those involved, to improve experience of care for patients, relatives and staff, and to reduce unnecessary transfers, needs to be evaluated using rigorous study designs including health economic analysis before wider implementation [30, 31]. 

## Conclusion

A number of factors significantly predicted conveyance of care home residents to hospital by ambulance, which included HCP referral and a higher NEWS2 score confirming that clinical severity significantly increased conveyance. Future interventions to prevent or address certain conditions, such as falls, or provide enhanced care in care homes may prevent some emergencies or reduce the risk of conveyance to hospital.

## Electronic supplementary material

Below is the link to the electronic supplementary material.

Supplementary Material 1

## Data Availability

The datasets generated and analysed during this study are not publicly available due to restrictions from the ethical approvals and data sharing agreements.

## Abbreviations

CI: Confidence Interval
ED: Emergency Department
HCP: Health Care Practitioner
MAR: Missing at random
NEWS2: National Early Warning Score 2
RRR: Relative Risk Ratio
