# Supplementary material for: Predictors of care home resident conveyance to hospital or referral to community pathways by a regional ambulance service attending medical emergencies: a retrospective cross sectional study
Source: Scand J Trauma Resusc Emerg Med. 2024 Nov 27;32:121. doi: 10.1186/s13049-024-01294-y (PMC11603973; doi:10.1186/s13049-024-01294-y)
Supplement: Supplementary file 1 — Supplementary Material 1 [file 13049_2024_1294_MOESM1_ESM.docx]

**Supplementary Information**

**Supplementary Table 1 Examples of conditions in each impression group category**

| **Cardiovascular** | **Respiratory** | **Trauma** | **Neurological** | **Mental Health** | **Gynaecological** | **Medical** | **Other** |
| --- | --- | --- | --- | --- | --- | --- | --- |
| Cardiac arrest | Asthma | Head, limb, back etc. Injury | Convulsion | Anxiety | PV bleeding | Allergic reaction | Fall- non injury |
| Cardiac chest pain | Chest infection | Cold / chemical exposure | Confusion | Dementia | Other gynaecological problem | Hypo/  hyperglycaemia | No apparent problem |
| Arrhythmia / Palpitations | Other respiratory problem | Suspected Neck of Femur | Headache | Depression |  | Palliative medical care | Transport only |

**Supplementary Table 2 Predictors of conveyance with imputed values and without imputed values (listwise deletion)**

|  | **Multiple imputation** | | **Listwise deletion** | |
| --- | --- | --- | --- | --- |
| **Baseline (Not conveyed)** |  | **Conveyed** |  |  |
|  | **RRR** | **95% CI** | **RRR** | **95% CI** |
| **Sex (Female)** | 1 | - | 1 | - |
| **Male**** | 1.07 | 1.03, 1.10 | 1.07 | 1.03, 1.10 |
| **Transgender** | 2.19 | 0.98, 4.87 | 2.50 | 1.06, 5.77 |
| **Age (under 60)** | 1 | - | 1 | - |
| **60-69** | 1.05 | 0.96, 1.14 | 1.05 | 0.96, 1.14 |
| **70-79**** | 1.09 | 1.03, 1.17 | 1.10 | 1.02, 1.18 |
| **80-89**** | 1.10 | 1.03, 1.17 | 1.11 | 1.05, 1.19 |
| **90-99** | 0.98 | 0.92, 1.04 | 0.99 | 0.93, 1.06 |
| **100 and over** | 0.61 | 0.54, 0.70 | 0.62 | 0.55, 0.71 |
| **Deprivation (Low)** | 1 | - | 1 | - |
| **High**** | 1.06 | 1.03, 1.09 | 1.06 | 1.02, 1.09 |
| **Rurality (Rural)** | 1 | - | 1 | - |
| **Urban** | 1.01 | 0.98, 1.05 | 1.02 | 0.99, 1.06 |
| **Impression Group (Other)** | 1 | - | 1 | - |
| **Medical**** | 8.93 | 8.46, 9.42 | 9.18 | 8.69, 9.69 |
| **Gynaecological**** | 23.84 | 15.37, 36.99 | 23.57 | 15.19, 36.57 |
| **Mental Health**** | 3.25 | 2.93, 3.60 | 3.30 | 2.97, 3.66 |
| **Neurological**** | 9.06 | 8.42, 9.75 | 10.26 | 9.51, 11.07 |
| **Trauma**** | 9.50 | 8.97, 10.05 | 10.17 | 9.59, 10.77 |
| **Respiratory**** | 6.81 | 6.35, 7.30 | 7.30 | 6.79, 7.84 |
| **Cardiovascular**** | 11.29 | 10.43, 12.22 | 11.51 | 10.62, 12.47 |
| **Call Category (1)** | 1 | - | 1 | - |
| **2**** | 1.48 | 1.39, 1.57 | 1.51 | 1.42, 1.60 |
| **3**** | 1.22 | 1.14, 1.30 | 1.23 | 1.15, 1.32 |
| **4**** | 13.28 | 11.48, 15.35 | 15.96 | 13.63, 18.68 |
| **5**** | 1.05 | 0.79, 1.41 | 1.05 | 0.78, 1.42 |
| **HCP**** | 15.37 | 13.41, 17.62 | 19.42 | 16.66, 22.63 |
| **First NEWS2**** | 1.23 | 1.22, 1.24 | 1.22 | 1.21, 1.23 |

**Supplementary Table 3 Comparison of patient demographic characteristics between care homes and the entire sample**

| **Demographics** | **Care home attendances** | **Non-care home attendances** |
| --- | --- | --- |
| **Median Age**** | 86 [78, 91] | 65 [39, 81] |
| **Gender** |  |  |
| **Female**** | 102,013 (59.99%) | 1,090,817 (51.92%) |
| **Male** | 67,959 (39.96%) | 1,009,063 (47.98%) |
| **Transgender** | 87 (0.05%) | 1,999 (0.10%) |
| **Ethnicity** |  |  |
| **White**** | 161,787 (98.13%) | 1,862,541 (93.00%) |
| **Black** | 1,264 (0.77%) | 40,584 (2.03%) |
| **Asian*** | 1,567 (0.95%) | 87,918 (4.39%) |
| **South East Asian** | 66 (0.04%) | 1,423 (0.07%) |
| **Traveller** | 0 | 3 (0.00%) |
| **Mixed Other** | 183 (0.11%) | 11,578 (0.58%) |

**Supplementary Table 4 Most frequent chief complaints attended by ambulance services in care homes**

| **Chief Complaint** | **Care Homes** | **Entire Sample (Baseline)** |
| --- | --- | --- |
| **NHS Pathways** | 26,924 (15.82%) | 489,775 (23.16%) |
| **Falls** | 19,216 (11.3%) | 108,841 (5.15%) |
| **Breathing problems** | 13,683 (8.04%) | 164,851 (7.80%) |
| **Unconscious/fainting** | 11,320 (6.65%) | 93,954 (4.44%) |
| **Escalate to yellow** | 9,110 (5.35%) | 48,623 (2.30%) |
| **Traumatic injury** | 8,122 (4.77%) | 44,874 (2.12%) |
| **Haemorrhage/Lacerations** | 7,498 (4.41%) | 61,839 (2.92%) |
| **Sick person** | 7,186 (4.22%) | 46,658 (2.21%) |
| **Chest pain** | 7,030 (4.13%) | 162,254 (7.67%) |
| **Pandemic flu** | 6,716 (3.95%) | 79,066 (3.74%) |
| **Healthcare professional admission** | 6,175 (3.63%) | 20,086 (0.95%) |
| **Convulsion/fitting** | 6,022 (3.54%) | 71,310 (3.37%) |
| **Stroke** | 5,694 (3.35%) | 47,251 (2.23%) |

**Supplementary Table 5 Physiological parameters as predictors of conveyance to hospital. The normal range as defined by NEWS2 scoring system was used as baseline category**

| Baseline (Not conveyed) |  | Conveyed |  |
| --- | --- | --- | --- |
|  |  | RRR | 95% CI |
| Respiratory rate (normal range 12-20 breaths/min) |  | 1 | - |
|  | Slightly abnormal 9-11 | 1.18 | 0.81, 1.72 |
|  | Abnormal 21-24** | 1.76 | 1.66, 1.86 |
|  | Severely abnormal lower end <8** | 0.16 | 0.13, 0.19 |
|  | Severely abnormal higher end >25** | 2.11 | 2.00, 2.24 |
| Heart rate (normal range 51-90 per minutes) |  | 1 | - |
|  | Slightly abnormal lower end 41-50** | 1.52 | 1.32, 1.74 |
|  | Slightly abnormal higher end 91-110** | 1.35 | 1.30, 1.41 |
|  | Abnormal 111-130** | 2.3 | 2.12, 2.5 |
|  | Severely abnormal lower end <40** | 0.62 | 0.52, 0.74 |
|  | Severely abnormal higher end >131** | 1.32 | 1.21, 1.44 |
| Blood pressure systolic (normal range 11-219 mmHg) |  | 1 | - |
|  | Slightly abnormal 101-110* | 0.94 | 0.89, 0.99 |
|  | Abnormal 91-100** | 1.16 | 1.07, 1.24 |
|  | Severely abnormal lower end <90** | 1.5 | 1.37, 1.64 |
|  | Severely abnormal lower end >220** | 0.56 | 0.52, 0.61 |
| Oxygen saturation (normal range 96-100%) |  | 1 | - |
|  | Slightly abnormal 94-95** | 1.19 | 1.14, 1.23 |
|  | Abnormal 92-93** | 1.6 | 1.49, 1.7 |
|  | Severely abnormal <91** | 2.38 | 2.24, 2.53 |
| Temperature (normal range 36-38 C) |  | 1 | - |
|  | Slightly abnormal lower end 35-36 | 1.00 | 0.95, 1.02 |
|  | Slightly abnormal higher end 38-39** | 1.61 | 1.45, 1.79 |
|  | Abnormal >39** | 0.71 | 0.66, 0.77 |
|  | Severely abnormal lower end <35** | 1.47 | 1.34, 1.62 |
